# Characterizing dengue transmission in rural areas: A systematic review

**DOI:** 10.1371/journal.pntd.0011333

**Published:** 2023-06-08

**Authors:** Olivia Man, Alicia Kraay, Ruth Thomas, James Trostle, Gwenyth O. Lee, Charlotte Robbins, Amy C. Morrison, Josefina Coloma, Joseph N. S. Eisenberg

**Affiliations:** 1 Department of Epidemiology, University of Michigan, Ann Arbor, Michigan, United States of America; 2 Department of Kinesiology and Community Health, University of Illinois, Urbana, Illinois, United States of America; 3 Institution for Genomic Biology, University of Illinois, Urbana, Illinois, United States of America; 4 Department of Anthropology, Trinity College, Hartford, Connecticut, United States of America; 5 Rutgers Global Health Institute, Rutgers, The State University of New Jersey, New Brunswick, New Jersey, United States of America; 6 Rutgers Department of Biostatistics and Epidemiology, School of Public Health, Rutgers, The State University of New Jersey, New Brunswick, New Jersey, United States of America; 7 Department of Pathology, Microbiology, and Immunology, School of Veterinary Medicine, University of California, Davis, Davis, California, United States of America; 8 Division of Infectious Diseases and Vaccinology, School of Public Health, University of California, Berkeley, Berkeley, California, United States of America; Louisiana State University, UNITED STATES

## Abstract

Dengue has historically been considered an urban disease associated with dense human populations and the built environment. Recently, studies suggest increasing dengue virus (DENV) transmission in rural populations. It is unclear whether these reports reflect recent spread into rural areas or ongoing transmission that was previously unnoticed, and what mechanisms are driving this rural transmission. We conducted a systematic review to synthesize research on dengue in rural areas and apply this knowledge to summarize aspects of rurality used in current epidemiological studies of DENV transmission given changing and mixed environments. We described how authors defined rurality and how they defined mechanisms for rural dengue transmission. We systematically searched PubMed, Web of Science, and Embase for articles evaluating dengue prevalence or cumulative incidence in rural areas. A total of 106 articles published between 1958 and 2021 met our inclusion criteria. Overall, 56% (*n* = 22) of the 48 estimates that compared urban and rural settings reported rural dengue incidence as being as high or higher than in urban locations. In some rural areas, the force of infection appears to be increasing over time, as measured by increasing seroprevalence in children and thus likely decreasing age of first infection, suggesting that rural dengue transmission may be a relatively recent phenomenon. Authors characterized rural locations by many different factors, including population density and size, environmental and land use characteristics, and by comparing their context to urban areas. Hypothesized mechanisms for rural dengue transmission included travel, population size, urban infrastructure, vector and environmental factors, among other mechanisms. Strengthening our understanding of the relationship between rurality and dengue will require a more nuanced definition of rurality from the perspective of DENV transmission. Future studies should focus on characterizing details of study locations based on their environmental features, exposure histories, and movement dynamics to identify characteristics that may influence dengue transmission.

## Author summary

Dengue is a widespread mosquito-borne viral disease with a large global burden. Previous research has focused on transmission in urban areas and only recently has been identified in rural populations. This systematic review that aims to (1) identify rural indicators used in current studies of dengue transmission; (2) characterize dengue seroprevalence and incidence in rural areas; and (3) summarize those studies that compare the risk of dengue in rural and urban locations.

## Introduction

Dengue is a significant public health challenge with roughly half of the world’s population living in endemic countries and more than 3 billion people at risk [1,2]. It is estimated that 96 million symptomatic dengue cases occur per year, which is likely an underestimate due to underreporting and misdiagnosis, particularly in countries with insufficient surveillance systems or lack of diagnostic capacity [3]. Urban centers have historically been thought to be at elevated risk for dengue due to (i) increasing population density that allows vectors to transmit dengue virus (DENV) to large pools of susceptible individuals without flying long distances [4–8]; (ii) increased human movement to and from hot spots of transmission; (iii) infrastructure failure (i.e., breakdown of water and waste management systems); and (iv) human activities leading to an abundance of urban-associated *Aedes aegypti* oviposition sites such as abandoned tires, vessels that can hold small amounts of water, and larger containers to store water for household use [9]. These factors favor vector abundance within urban centers that have the population and other ingredients for sustained arboviral transmission.

Dengue research has therefore largely (but not exclusively) focused on virus transmission in urban areas [2,10–16]. However, an increasing number of studies have described high levels of DENV infections in rural populations [4,17–19]. Some have postulated that the increase in rural DENV transmission is due to the increase in human travel [20]. As transportation infrastructure has developed worldwide, travel between urban and rural areas as well as human mobility between remote regions has increased, facilitating virus spread into and out of rural settings [21]. Additionally, some of the urban features that are commonly considered to drive risk may increasingly be present in more rural areas, including higher population density and habitats suitable for *Ae*. *aegypti* development. Finally, because DENV infections are often asymptomatic [1], the burden of infection may be underestimated in poor or rural settings where surveillance systems are less developed. It is increasingly recognized that rural DENV transmission may be crucial for maintaining serotype diversity in populations and may also facilitate disease reemergence [22–24].

Definitions of what is rural vary widely in the dengue literature. For example, researchers have defined rural environments based on population size [25], population density [4,17, 25–27], housing density [28], infrastructure or surface cover type (impervious surfaces, vegetation) [29,30], access to urban areas or distance to a road/urban center [31,32], environmental changes (including changes to landscapes, rural production systems, climate, land use, and transportation infrastructure) [27,32,33], or agricultural practices [25]. A definition useful for surveillance and mitigation may be a function, subset, or combination of these factors and may vary by region. Our overall goal is to summarize rurality indicators used in current epidemiological studies of DENV transmission given changing and mixed environments. To achieve this goal, we conducted 3 subanalyses: (1) to review how authors defined rural and the mechanisms for rural transmission; (2) to summarize those studies that compare the incidence of rural dengue to urban estimates; and (3) to assess dengue seroprevalence and incidence in rural areas.

## Methods

### Study registration

This protocol has been registered with the international prospective register of systematic reviews (PROSPERO) (registration number 92243). The Preferred Reporting Items for Systematic Reviews and Meta-analyses (PRISMA) Statement guided the conduct of this review [34].

### Search strategy

Literature search strategies were developed under the guidance of an information specialist from the Health Science Library at the University of Michigan (GR). Medical Subject Headings (MeSH) and text word searches related to dengue and urbanicity were used in PubMed, Embase, and Web of Science. The following MeSH and keyword terms were used in PubMed (*n* = 324), Embase (*n* = 380), and Web of Science (*n* = 30) articles: (“rural population” or “rural health” or “urbanization”) AND (“dengue” or “Dengue virus”). The search was conducted on 18 November 2021.

### Eligibility

All studies and official reports published in English or Spanish were eligible for inclusion based on the criteria below. We did not exclude articles based on study design or quality, as the strength of evidence from each study was evaluated separately using an adapted version of the Newcastle–Ottawa Scale (NOS) [35]. We also did not limit our study by year of publication. Because there is no international consensus for the definitions of “rural” and “urban” [36–38], the United Nations recommends following definitions based on regional perceptions [38]. Therefore, we considered study sites to be “rural” or “urban” based on the original authors’ designations. It follows that we only considered “urban” study sites if authors had directly compared them to a “rural” location. An alternative approach to our eligibility criteria would be to classify studies based on a predefined list of specific rural characteristics; however, this could bias our review because not all features of rural environments may be reported by all papers.

### Inclusion criteria

We included studies that meet one or more of the following criteria:

(1) Authors describe the study site as “rural,” or a similar descriptor, or describe a rural–urban continuum in the title or abstract and one or more of the following:
  (a) mentions at least one positive or probable dengue case; or
  (b) describes dengue seroprevalence; or
  (c) describes an outbreak of dengue; or
(2) Studies or official reports of dengue incidence, prevalence, or outbreaks in humans of a larger geographic area where rural estimates could be extracted. For example, the Pan American Health Organization’s Health Information Platform for the Americas provides estimates at fine geographic resolution such that rural estimates can be isolated.

### Exclusion criteria

We excluded studies if (1) the study did not provide human outcome data on dengue, including all studies that only mention mosquitoes and/or zoonotic transmission without any description of dengue incidence or prevalence; (2) news articles, commentaries, factsheets, or other source formats that do not provide dengue outcome data; or if (3) the authors described location as “a city” or “urban” in the title or abstract; or (4) if the authors mention the name of the study location and the location has a population greater than 100,000 without providing dengue estimates from a comparable rural study location. The addition of a 100,000-population cutoff was used to exclude large cities, reducing the number of irrelevant studies to be screened.

### Data collection process

Two reviewers (OM and RT) independently extracted data from the included sources on study population, methodology, and reported dengue outcomes. Reviewers resolved disagreements by discussion and a third reviewer (ANMK or JNSE) decided on any unresolved disagreements. See supporting information for more details on the data collection and quality assessment. In brief, the quality of each study was ranked using an adapted version of the NOS (see supporting information for more details).

### Analysis

All studies that met our inclusion criteria were considered for qualitative review, including articles that described a relationship but did not provide a numeric point estimate. While we used our NOS subscale to determine which of our hypothesized rural characteristics were also mentioned by study authors, we also extracted characteristics mentioned by study authors separately to create a narrative summary of what has been found in the literature that was unbiased by our prior expectations. In addition to characteristics used to define rurality in the articles included in this review, we also separately extracted authors’ hypothesized mechanisms for their measured rural incidence.

Studies were considered for the quantitative analysis if authors provided information about cases that could be approximated to dengue incidence, prevalence, or seroprevalence estimates. In order to include a larger variety of studies with varying research questions, we hand calculated estimates when necessary.

When studies could be grouped by comparable definitions of the exposure and outcome, we conducted a meta-analysis using a random effects model to quantify the overall measure of association for objectives (2) and (3). For rural dengue studies, we also considered how reported incidence might be influenced by key effect modifiers that varied across studies, including population size and the method of data collection.

## Results

**Fig 1:**
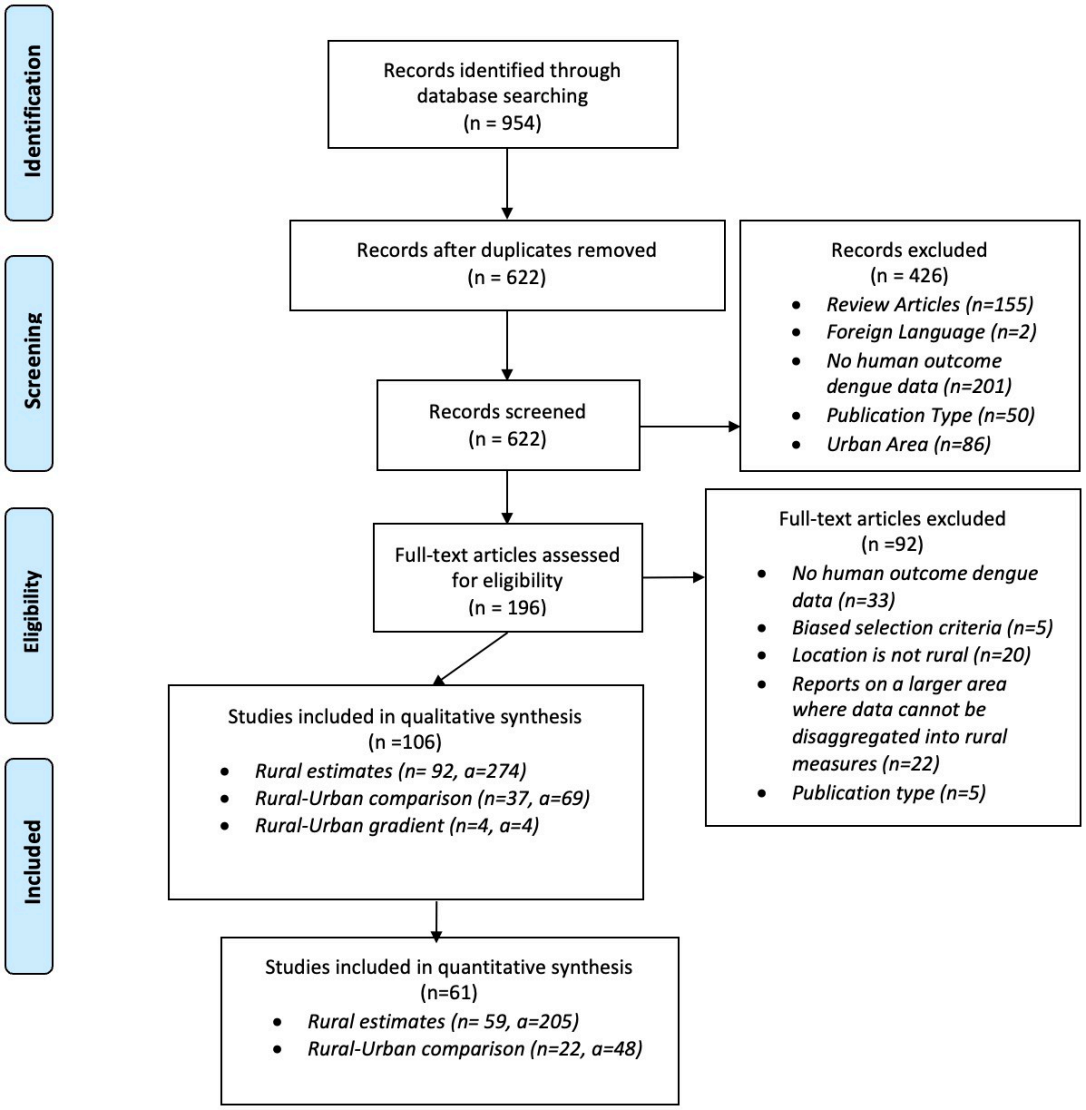
PRISMA diagram of articles included in our analysis where “n” represents the number of articles and “a” represents the number of estimates. *From*: Moher D, Liberati A, Tetzlaff J, Altman DG, The PRISMA Group (2009). *P*referred *R*eporting *I*tems for *S*ystematic Reviews and *M*eta-*A*nalyses: The PRISMA Statement. PLoS Med 6(7): e1000097. doi:10.1371/journal.pmed1000097.

**Fig 2:**
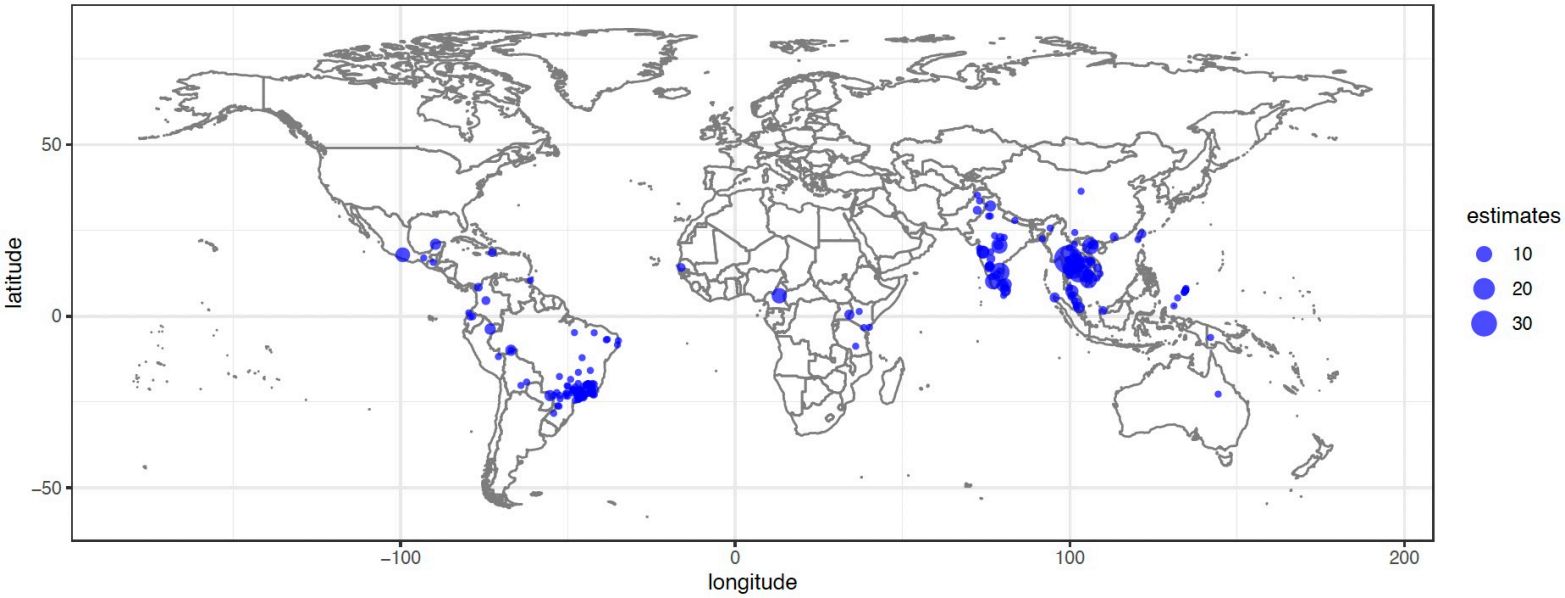
Map of study locations. The size of dot corresponds to the number of estimates. The basemap is publicly available at https://www.naturalearthdata.com/downloads/50m-raster-data/50m-bathymetry/.

A total of 106 studies and 347 estimates were included in our qualitative synthesis [4–9,19,25,39–135] (Fig 1). Most estimates used data collected in tropical regions of Southeast Asia, Latin America, and Africa (Fig 2). These studies were divided into 2 categories: (1) articles that presented rural–urban comparison estimates (*n* = 37) [4–7,9,25,39,42–44,49,53,59–62,65,69,72,75,78,83,85,86,88,93,95–97,101,109,119,120,125,126,133,135] and (2) articles that presented rural estimates (*n* = 92) [4–9,19,25,40–71,73–78,80–82,84,85,87,89–94,97–100,102–109,111–119,121,122,124–132,134,135]. In addition, we identified 4 studies that modeled dengue along rural–urban gradients [79,82,123,134]. Of these, three observed higher dengue incidences in urban compared to rural areas [32,39,41], whereas one found the opposite [40]. We did not consider the gradient studies any further due to noncomparable analysis methods.

For the qualitative analysis, studies were assessed for reported definitions of rurality and author hypothesized mechanism for rural dengue transmission.

Of the 106 studies that met our inclusion criteria, 45 articles were excluded from the quantitative synthesis (Fig 1) [19,39,40,42,45,47,49,52–56,60,66,67,71,72,76,80,83,85–88,90,95,96,101,104,107,110,111,114,115,117,118,120,122,124,125,127–129,133,134]. Most articles were excluded because they lacked information on variance (73.3%, *n* = 33) or they did not report the underlying population (e.g., they only reported the proportion of fever cases, 48.9%, *n* = 22). Other reasons for exclusion include biased sampling methods and incomparable case information (e.g., a rise in cases after natural disasters). Articles included had data collection dates ranging from 1987 to 2016.

### Objective 1: Qualitative analysis (*n* = 106)

#### Rural definitions

For all studies included in our qualitative synthesis, we evaluated the rural definitions and study site descriptions used by the authors (Table 1). One-third of the articles did not provide justification for classifying their study site as rural (34.9%, *n* = 37) [4,5,40–42,47,49,52,56,65–67,72,75,77,78,87,89–93,95–97,107,110,111,113,118,120,121,124,125,127,129,130,132]. The remaining 69 studies provided a wide variety of rural definitions. We grouped these definitions into 4 broad categories: population, environment and land use, relationship to urban areas, and miscellaneous, which we further divided into 13 specific subcategories. An article often used a definition that allowed them to be placed in more than 1 category. When definitions related to population characteristics were used, it was more common for authors to consider population size (57.1%, *n* = 28) [8,19,44,46,48,50,61,63,64,68,70,74,76,82,83,86,94,100,102,103,105,106,108,109,116,119,122,128] than population density (28.6%, *n* = 14) [6,8,19,48,73,76,83,100,101,103,108,109,115,134] or housing density (14.3%, *n* = 7) [19,44,61,63,99,126,131]. When authors used environmental and land use definitions of rural, they tended to use various descriptors. Examples include terrain, such as “tropical forests” or “rice fields,” proximity to increased wildlife biodiversity (52.5%, *n* = 21) [45,48,51,54,55,57,69,71,81,88,99,100,105,106,114,115,117,119,122,134,135] or agricultural practices, including the types of crops and livestock, (47.5%, *n* = 19) [8,43,45,57,58,62–64,68,70,71,83,100,104,114,115,119,128,134]. If rural was defined in relation to urban centers, authors commonly used direct comparisons and considered locations to be rural because (a) they were not urban (50.0%, *n* = 15) [7,9,25,39,43,53,59,62,69,79,83,85,86,109,133]; (b) the distance from an urban location was above a threshold (36.7%, *n* = 11) [6,50,54,55,63,64,80,82,98,120,124]; or (c) travel time to an urban location was above a threshold (13.3%, *n* = 4) [60,64,82,84]. Other miscellaneous definitions of rural included quality of transportation systems [58,60,64,84,106,112,115], water access or usage [60,64,108,119], quality of housing or roads [60,88,115,119], socioeconomic status [68,73,99,126], or a country-wide classification system [62,126].

**Table 1:** Characteristics used by authors in their rural definitions (106 studies included 177 cited characteristics; 37 studies had no definition of rural and many articles used more than one definition).

Definition of Rural	No. of Articles	No. of Articles (% within category)
Undefined	37	37 (100%)
*Population Characteristics*	49	
Population		28 (57.1%)
Population density		14 (28.6%)
Housing density or number of households		7 (14.3%)
*Environmental and Land Usage Characteristics*	40	
Terrain characteristics and presence of wildlife		21 (52.5%)
Agriculture		19 (47.5%)
*Relative to Urban Areas*	30	
Direct comparison to urban*		15 (50.0%)
Distance from urban area		11 (36.7%)
Travel time to urban area		4 (13.3%)
*Miscellaneous Characteristics*	21	
Description of transportation systems		7 (33.3%)
Limited water access or use		4 (19.1%)
Socioeconomic status		4 (19.1%)
Description of housing or road conditions		4 (19.1%)
Country-wide classification system		2 (9.5%)

*Studies in the “direct comparison to urban” category defined locations as rural because they did not meet the authors’ criteria for an urban center.

#### Reported modes of transmission

We also searched each of the 106 articles used in this review for possible modes of DENV transmission in the rural study sites. Thirty (28.3%) articles did not discuss how transmission occurred (Table 2). Of the remaining 76 (71.7%) articles, one or more modes of transmission were suggested by study authors, most of which were included in author-specified definitions of rural areas (Table 1). We placed the articles into 19 general transmission categories (Table 2) that fell into 4 groups: (1) human population (travel, movement, and population size); (2) urban infrastructure (water storage, improper water management, proximity to roads, increasing infrastructure, and unspecified urbanization, mentioned by 30 articles); (3) vector and environmental factors (mentioned by 34 articles); and (4) other mechanisms (emergence in new areas, underreporting, low levels of prior immunity, and biological variability, mentioned by 18 articles). Some articles also mentioned the importance of local seroprevalence patterns, which define susceptibility of local populations, and disease underreporting associated with poor or rural areas.

**Table 2:** Hypothesized mode of dengue virus transmission suggested in articles included in this systematic review.

Hypothesized mode of transmission	No. of Articles	References
***Human population***	**42**	
Travel to urban centers	16	[6,7,46,48,49,57,63,69,79,80,82,108,110,119,120,123]
Human movement	18	[19,25,43,48,55,57,59,62,68,69,91,101,106,113,121,122,126,131]
Increasing population density or size	20	[4–6,9,25,39,43,45,55,69,84,93,100,109,110,121,123,125,134]
Introduction through travel, not sustained	3	[39,78,82]
***Urban infrastructure***	**30**	
Increases in water storage	15	[6,9,43,44,49,53,62,64,76,80,84,96,123,127]
Insufficient waste management (increasing container habitat availability)	6	[9,39,43,54,119,123]
Proximity to roads	5	[6,25,101,106,110]
Building infrastructure	7	[9,25,39,75,81,84,119]
Unspecified urbanization	10	[5,9,43,72,81,93,94,109,127,128]
**Vector and environmental mechanisms**	**34**	
Land use and agriculture	7	[59,68,76,96,106,108,134]
Exposure to natural environments	11	[43,51,54,58,68,69,84,88,95,101,134]
Sylvatic cycle	3	[51,63,69]
Climate Change	5	[60,68,93,121,134]
Other vector related mechanisms (i.e., alternate vectors, vector densities, vector spread)	22	[43–45,49,53,58–60,62,64,68,69,72,75,76,93,95,101,110,114,126]
**Other modes of DENV transmission**	**18**	[59,71,72,75,77,80,82,95,98,104,109,111,112,123,125,127,131,135]
Prior underreporting or data collection limitations	7	[59,75,83,95,109,117,123]
Emergence in new area	9	[71,72,80,98,104,111,112,127,135]
Emergence of a new serotype	2	[82,98]
Higher susceptibility in rural areas (due to low prior exposure)	2	[82,131]
Biological variability uncaptured by standard variables	1	[74]
**None**	**30**	[40–42,47,50,52,56,61,65–67,70,73,74,77,87,89,90,92,97,99,102,103,107,115,116,124,130,132,133]

### Objective 2: Rural–urban comparison

**Fig 3:**
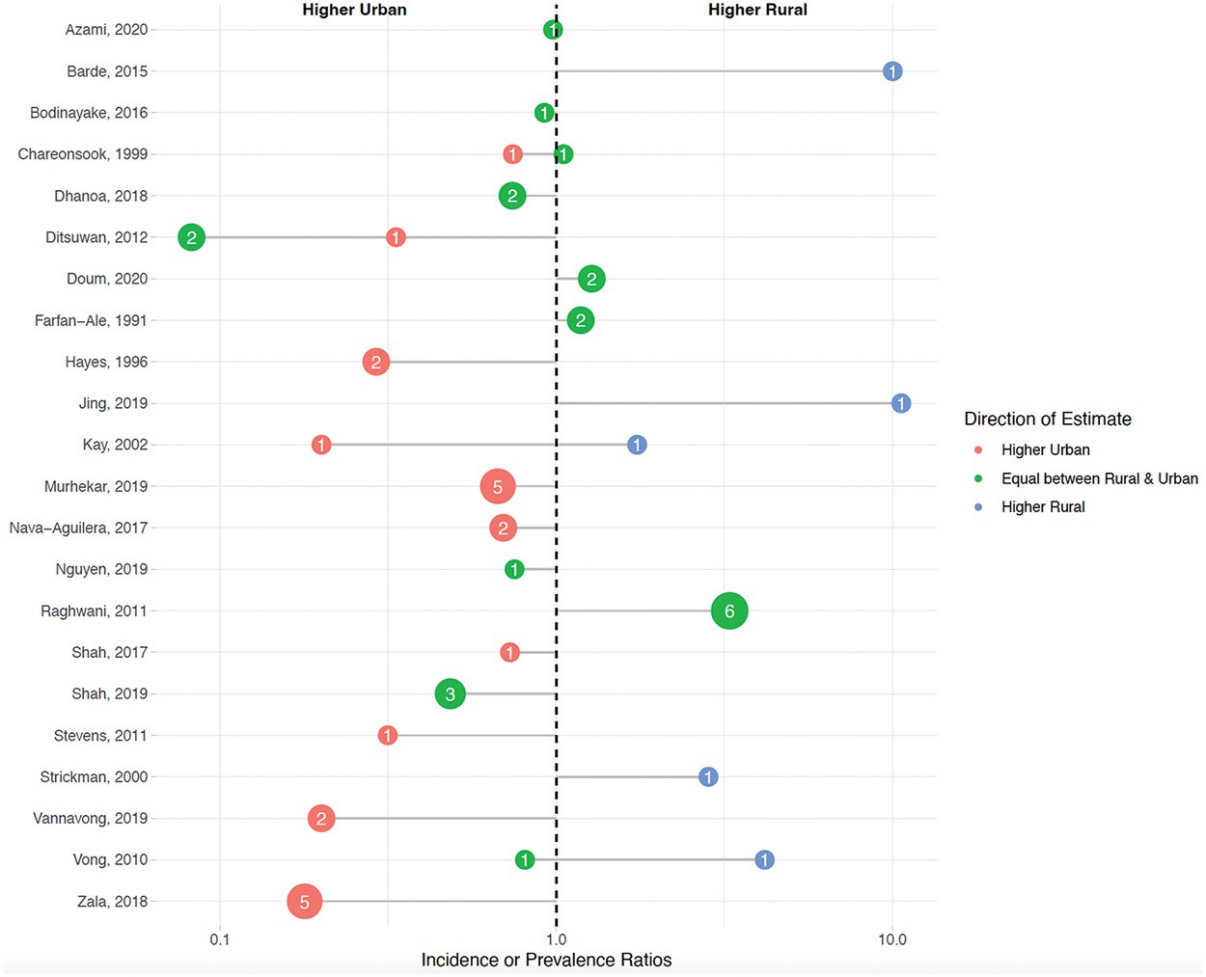
Distribution of rural to urban dengue incidence and prevalence ratios. Each dot represents the average incidence or prevalence ratio grouped by direction of the estimate. The size and number of each dot represents the number of estimates. Red-colored estimates signify that incidence/prevalence was higher in urban areas; green-colored estimates signify that incidence/prevalence was equal between rural and urban areas (i.e., had confidence intervals that crossed the null value); and blue-colored estimates signify that incidence/prevalence was higher in rural areas.

We identified 48 estimates from 22 articles comparing rural to urban dengue estimates [4,6,7,9,25,43,44,49,53,59–62,65,69,75,78,93,97,109,126,135]. The rural–urban comparison studies had an average NOS quality ranking of 3.5, with a standard deviation of 1.1. Eighteen articles and 40 estimates reported values that could be approximated as incidence ratios [4,6,7,9,44,49,53,60–62,65,69,78,93,97,109,126,135], and 7 articles with 8 estimates reported prevalence or seroprevalence ratios [25,43,59,62,65,69,119]. While some of these articles used different study designs, the ratios from these articles could be compared across studies to assess rural versus urban patterns of disease. Overall, 56% (*n* = 22) of these estimates found that dengue was as high or higher in rural areas (Fig 3) [4,6,7,9,25,43,44,49,53,59,61,62,65,69,75,78,93,97,109,119,126,135]. This pattern of similar or higher rural dengue compared with urban dengue was similar regardless of population size or the method of data collection (active versus passive). Several studies assessed dengue cases overtime, showing either that values in rural and urban areas eventually became more similar or that cases were higher in urban populations after years of circulation [4,6,53,61,78].

### Objective 3: Rural dengue

We identified 59 articles (n) with 205 estimates (a) estimating dengue in rural areas [4–9,25,41,43,44,46,48,50,51,57–59,61–65,68–70,73–77,81,82,84,89,91–94,97–100,102–104,106,108,109,113,116,119,121,126,130–132,135]. These estimates were further categorized into measurements of seroprevalence (*n* = 18, a = 37) [25,43,46,50,51,57–59,62,65,68,69,75,81,91,93,109,119], incidence (*n* = 32, a = 119) [4–9,41,48,61,62,65,69,70,73,76,77,89,92,99,100,102,104,108,109,113,116,121,126,130–132,135], prevalence (*n* = 5, a = 8) [74,97,106,131,132], and outbreak studies (*n* = 8, a = 40) [44,63,64,82,84,94,98,103].

#### Seroprevalence

Studies that looked at rural dengue seroprevalence tended to have higher NOS scores (mean = 4.2, standard deviation = 0.6) when compared to the other rural result subgroupings. Among these studies, seroprevalence was not statistically related to population size (*p* = 0.1832). Five studies that stratified rural seroprevalence by age show increasing seroprevalence among children over time [25,57,65,109,119] (S1 Fig). Several seroprevalence articles surveyed large geographic areas, one of which could be divided into smaller villages.

#### Rural incidence

Studies that looked at rural incidence had lower NOS scores (mean = 2.5, standard deviation = 1.1) when compared to other rural result subgroupings. Most of these were community-based studies that included active case finding rather than passive surveillance. The average follow-up time for active surveillance studies was 381.2 days [5–7,62,65,69,70,73,78,99,100,109,126,130–132] compared to the average follow-up time of 1,118.7 days for passive surveillance studies [4,8,9,41,48,61,76,77,89,92,102,108,113,116,121,135]. Estimates collected from passive methods (i.e., hospital referrals) reported lower incidence rates than studies that used more active case finding (i.e., cohort, community surveillance). Passive data collection studies (*n* = 16, a = 63) had a pooled annual incidence estimate of 142 cases per 100,000 [95% CI: −537, 822, range: 0, 2,133] (S2 Fig). Active data collection studies (*n* = 16, a = 56) had a pooled incidence estimate of 2,469 cases per 100,000 [95% CI: 1,471, 3,466, range: 0, 17,066] (S3 Fig). For some studies, the incidence of rural dengue appeared to be increasing over time, but this trend was inconsistent. Moreover, while several articles presented dengue incidence for consecutive years [4,6,61,70,78,121], they did not report information on the predominant serotype (DENV1–4). If the same serotype was circulating during sequential years, yearly estimates may capture multiple or incomplete transmission cycles. Rural incidence appeared to be similar regardless of population size.

#### Rural prevalence

Studies that collected information on rural prevalence had low NOS quality scores (mean = 2.5, standard deviation 0.8). Due to the small number of articles (*n* = 5, a = 8) and variable definitions of dengue (e.g., self-reported household dengue or prior history of dengue), we did not further evaluate the effect of potential drivers of rural prevalence [74,97,106,131,132].

#### Rural outbreaks

Articles that evaluated outbreaks of dengue in rural areas had an average NOS quality score of 2.2 (standard deviation 1.1). There were 8 articles on outbreaks with 40 estimates [44,63,64,82,84,94,98,103]. Further assessment of rural outbreaks was difficult due to variations in follow-up time, sampling methods, and definitions of probable dengue infections. For example, one article only considered positive cases during a 2-week period [103], while another study only considered cases with positive neurologic side effects (e.g., altered mental status) [63]. Additionally, it is unclear if data collection spanned the entirety of the outbreak. Although some articles stratified by age group [98,103], stratifications were inconsistent.

## Discussion

In this review, we compiled literature on rural dengue with 3 objectives in mind: (1) to identify definitions and mechanisms of rural dengue transmission; (2) to summarize those studies that compare dengue risk in rural areas with urban areas; and (3) to assess the burden of dengue in rural areas. Though 37% of 106 studies did not define the term “rural” at all, the remaining studies most commonly defined rural dengue based on population characteristics and environmental and land usage characteristics (Table 1). The overall level of incidence in rural areas was commonly related to human population, the level of local infrastructure, and environmental and vector characteristics (Table 2) (Objective 1). Based on studies comparing urban and rural dengue, we find evidence that the risk DENV transmission in rural areas is substantial and is often similar to or sometimes higher than the risk in urban areas (Fig 3). This suggests that rural regions might be an important source of dengue cases (Objective 2). Based on those articles focusing on rural areas alone, burden was nontrivial, and some data suggested that rural dengue incidence might be increasing over time (S1 Fig) (Objective 3).

### Objective 1: Identify definitions and mechanisms of rural dengue transmission

Study authors used a variety of rural definitions based on political definitions, population characteristics, environmental and land usage patterns, distance/travel time to urban centers, and transportation systems (Table 1). Underlying these study definitions of rurality are proposed or assumed mechanisms and factors related to rural DENV transmission (Table 2). The varied definitions and wide range of proposed mechanisms highlight complexities in the understanding of rural DENV transmission dynamics and ability to compare results across studies. This finding is consistent with prior research that has shown that definitions of “rural” vary across regions [36–38] and that multiple features beyond those, which have been typically classified as “rural” characteristics, tend to impact risk.

While many features were only mentioned by a few authors, environmental exposures and local infrastructure were mentioned frequently as both definitions of rural areas (Table 1) and subsequent drivers of dengue incidence. However, in most instances, rural drivers of DENV transmission appeared to be locally specific and did not show a relationship at aggregate levels. For example, we did not observe a quantifiable relationship between population size and rural dengue seroprevalence. This suggests that population size may be a driver of DENV infection in some rural locations but is not sufficient to explain the existing global trends in the literature. Thus, population may not be an adequate proxy for rurality alone. Local differences in population size might be more useful in defining geographic patterns of risk rather than defining absolute transmission levels globally. Unfortunately, we were not powered to assess the impact of population size for incidence studies. Follow-up studies that assess the impact of population size might provide more insight on this relationship.

Most articles we reviewed also described changing features of rural environments associated with increasing transmission. These include (i) a higher rural incidence among politically defined rural areas with multiple “urban” characteristics [6,44,78]; (ii) improvements in infrastructure and vector control mechanisms in urban centers, such as a regular water supply [6,62,127], which shift the disease burden further towards more rural settings without parallel improvements in infrastructure; and (iii) other changes to rural environments that may promote DENV transmission, including the increased introduction of plastics [9,44,49,119], the proliferation of new roads [6,25,106,110], and changes in land use [59,76,106,108]. These changing rural environments and evolving ways in which humans interact with their environments may contribute to the differential DENV transmission across various rural and urban settings.

### Objective 2: Compare dengue risk in rural and urban areas

We found that the risk of DENV transmission in rural areas is often as high or higher than the risk in urban areas (Fig 3). While many papers included in this review suggested that rural dengue is an emerging challenge, our review also illustrates the complexity of dengue in rural regions, showing that dengue transmission is not best explained by a rural versus urban dichotomy. As rural transmission increases, the articles included in our review suggest that ongoing urbanization may create a feedback loop where new land and network development may bring rural sectors in closer proximity to urban sectors and increase movement of humans, goods, and services between urban and rural areas. The role of urbanization on dengue transmission in our review has specifically been attributed to regional development, such as the construction of new roads [6,25] and increasing transportation systems [6,7,49,53,69,78,135], which may allow importation of infected individuals [121] and the expansion of population densities or sizes [6,9,25,58,100]. This has also resulted in communities encountering new challenges that may potentiate DENV transmission, such as inadequate waste management systems [9,119] and changing water storage practices [6,9,76]. Other changes, such as to housing infrastructure [119] or land use [58,59,68,76,108], may contribute to increasing vector densities [76] through further changes to breeding sites and human exposure to the environment. These factors may create environmental patches of high and low risk condition that cross the politically defined rural and urban sectors [21].

### Objective 3: Assess dengue burden in rural areas

While our results are insufficient to confirm that incidence is increasing in rural areas, several other lines of evidence from this review also support this conclusion. Four studies that stratified rural seroprevalence by age show increasing seroprevalence among children over time [57,109,119] (S1 Fig). This implies that the average age of first infection may be decreasing and rural incidence may be increasing over time. Some studies suggested an increasing incidence over time; however, comparison across regions was difficult as DENV transmission can cycle and overall patterns may vary depending on the time scale used. Generally, longer time scales would be less likely to be susceptible to this bias. Thus, future studies may obtain more reliable estimates when using longer follow-up studies.

Additionally, the incidence of dengue we observe here may be related to a true increase or result from other factors. For example, improved surveillance systems may lead to improved case reporting, even as incidence remains unchanged. We find that relatively few studies provided sufficient information to distinguish between these 2 possibilities. More detailed laboratory data that combine seroprevalence and prospective incidence data from cohort studies could help disentangle these 2 mechanisms. The methods used in case ascertainment may have a large impact on an observed association with dengue in the literature. For example, many articles captured by our review come from passive hospital-based surveillance systems [4,6,9,49,53,61,78,135], which may disproportionately underestimate rural cases. These systems fail to identify people who do not seek healthcare and those with milder presentations [44,78], which may be amplified by transportation challenges in more remote or poorer areas. As expected, the overall incidence of dengue for the articles in our review was generally lower among studies using passive surveillance systems compared with studies with more active case finding methods (S2 and S3 Figs).

Other factors contributing to inconsistent DENV rural estimates may include study size, varied definitions of rurality, and local differences in mechanisms. These inconsistent estimates may be related to the sample sizes and aggregation used in many studies. Many studies had small sample sizes, making their quantitative estimates less reliable. Moreover, several studies assessed dengue seroprevalence in large rural areas or combined rural estimates from several different villages, resulting in relatively large rural populations with variations in cases between specific sites [58,119]. While such studies might be more representative of rural populations in general, pooling communities in this way made it difficult for us to quantify the impact of individual community size on dengue risk.

While it seems unlikely that isolated rural areas captured by the articles in our review have a sufficient population to sustain endemic levels of dengue, some authors hypothesized that transportation networks among rural communities resulted in population dynamics that mimic urban centers [69]. Others have noted the role of a sylvatic cycle and exposure to natural environments on rural DENV transmission [58,59,68,69]. Areas with close contact to heavily vegetated areas and primates may create the opportunity for spillback sylvatic transmission and periodic reintroduction of arboviruses. While none of the articles reviewed found definitive evidence of sylvatic DENV transmission to humans [69,81], some have discussed how changes in rural land use may increase the potential for human exposure to the existing natural transmission cycles [58,59,68]. If rural dengue is independently sustained by larger networks of rural populations or through reintroduction by sylvatic cycles, it is possible that rural circulation may be sufficient to maintain a reservoir. When immunity in urban centers is high and growing transportation networks may facilitate the reintroduction of DENV into urban centers once population immunity declines.

## Limitations

The primary limitation of this review was the inconsistent quality of surveillance systems in the literature and a lack of longitudinal data, which made it difficult to assess changes in dengue burden over time. Secondly, while our inclusion criteria focused on studies in rural sites including urban areas only if they were compared directly with rural sites by the same research group, this strategy a large proportion of available incidence data available from urban sites, potentially limiting our ability to compare incidence in urban and rural areas more generally. Moreover, given that there is no international consensus on what is considered “rural” or “urban,” we used regional definitions, as recommended by the UN [38]. Thus, a study in one region reporting rural dengue might have been classified as urban in another context. We are hopeful that the detailed data captured in this review can help the field move towards standardized definitions in future studies.

## Conclusions and public health implications

There is a pressing need to better understand the changing and potentially increasing burden of DENV transmission in rural areas. While population size may be related to dengue risk, our results suggest that understanding the environmental and infrastructure features that drive risk may be just as important—if not more important—than absolute population size. As population size continues to increase in rural areas, our review suggests that infrastructural improvements are needed to minimize population vulnerability. For example, reliable water systems and safe waste disposal might help mitigate concerns about growing dengue incidence by limiting breeding sites. The increases in transportation networks may connect smaller rural regions, increasing the effective population size. Quality surveillance systems to track dengue incidence in rural areas can help identify new surges in population risk that might accompany changing infrastructure such as transportation. In the absence of such interventions, the often underresourced rural regions may play a major role in the spread of dengue infection, serving as sources of new infections as well as sinks to introduced from other regions without being noticed. Because these same infrastructure features change with ongoing urbanization, a feedback loop may be created that drives incidence to continue to increase in rural areas. Better identifying key features of dengue risk in rural locations will promote surveillance and support the development of intervention strategies that are most appropriate in this context.

## Supporting information

S1 Fig Rural dengue seroprevalence (%) in children over time. Each association is from a different study.
(DOCX)

S2 Fig Yearly incidence per 100,000 from studies that used passive methods to collect rural dengue data. (DOCX)

S3 Fig Yearly incidence per 100,000 from studies that used active methods to collect rural dengue data. (DOCX)

S1 Table Adapted Newcastle–Ottawa Scale (NOS) criteria for systematic reviews. (DOCX)

S1 Text Supplementary text. (DOCX)
