# Supplementary material for: Characterizing dengue transmission in rural areas: A systematic review
Source: PLoS Negl Trop Dis. 2023 Jun 8;17(6):e0011333. doi: 10.1371/journal.pntd.0011333 (PMC10249895; doi:10.1371/journal.pntd.0011333)
Supplement: S1 Fig — Each association is from a different study. (DOCX) [file pntd.0011333.s001.docx]

| **S1 Figure:** Rural dengue seroprevalence (%) in children over time. Each association is from a different study. |
| --- |
| **** |
