# Supplementary material for: Characterizing dengue transmission in rural areas: A systematic review
Source: PLoS Negl Trop Dis. 2023 Jun 8;17(6):e0011333. doi: 10.1371/journal.pntd.0011333 (PMC10249895; doi:10.1371/journal.pntd.0011333)
Supplement: S3 Fig — (DOCX) [file pntd.0011333.s003.docx]

| **S3 Figure:** Yearly incidence per 100,000 from studies that used active methods to collect rural dengue data. |
| --- |
|   **Summary Estimate** |
