# Supplementary material for: Characterizing dengue transmission in rural areas: A systematic review
Source: PLoS Negl Trop Dis. 2023 Jun 8;17(6):e0011333. doi: 10.1371/journal.pntd.0011333 (PMC10249895; doi:10.1371/journal.pntd.0011333)
Supplement: S1 Table — (DOCX) [file pntd.0011333.s004.docx]

| S1 Table: Adapted Newcastle Ottawa Scale (NOS) criteria for systematic reviews |  |
| --- | --- |
| Criteria | Stars allocated |
| Selection: Representativeness of the base population |  |
| a) For studies focusing on a province/country, communities included in the study are representative of that province | * |
| B) For studies focusing on a smaller region within a country, communities included are representative of that region. | * |
| c) Single community is included or the population is not representative | -- |
| Comparability: if a study contains both urban and rural areas, surveillance or disease ascertainment activities were of similar strength in all locations. |  |
| A) Yes | * |
| B) No | -- |
| C) Does not consider a gradient | -- |
| Outcome: |  |
| 1. Was ascertainment of outcome adequate |  |
| A. Dengue confirmed by laboratory testing | * |
| B. Dengue determined by symptoms only | -- |
| C. Dengue determined by recall | -- |
| 2. Was asymptomatic infection captured? |  |
| A. Yes | * |
| B. No | -- |
| Exposure: |  |
| A. Authors clearly define exposure variable | * |
| B. Authors did not clearly define exposure variable | -- |
