# Supplementary material for: Characterizing dengue transmission in rural areas: A systematic review
Source: PLoS Negl Trop Dis. 2023 Jun 8;17(6):e0011333. doi: 10.1371/journal.pntd.0011333 (PMC10249895; doi:10.1371/journal.pntd.0011333)
Supplement: S1 Text — (DOCX) [file pntd.0011333.s005.docx]

**Supplementary Text**

**Data extraction details**

We extracted the following variables: study site and study population characteristics (location, demographics, sample size, community versus hospital-based), study design (randomized control trial, case report, cohort study, case-control study, etc.), time of the study (year and duration), season (high season, rainy season, temperature), outcome (dengue incidence, prevalence, or cumulative incidence), lab technique to confirm cases and/or clinical criteria used to confirm cases, method to evaluate probable cases (example: interviewers about previous dengue infections), and DENV serotype (DENV1-4). Our primary outcome was prevalence or cumulative incidence of dengue in humans in rural areas. Because authors commonly presented multiple findings per study that may contribute to our understanding of rural dengue virus spread, we often extracted multiple estimates per study with care to avoid extracting estimates that came from overlapping geographic regions and timeframes. For example, Strickman et al. collected data from a population of 10,000 living in 12 different villages. We extracted 12 different estimates corresponding to the 12 villages studied.

**Quality assessment**

The two reviewers rated the articles independently following an adapted version of the NOS method (S1 Table), commonly used in meta-analyses to evaluate the quality of observational studies. Studies could receive a maximum of 6 points from the NOS scale, corresponding to selection criteria, comparability, outcome, assessment of asymptomatic infections, and clear exposure criteria. The original NOS method has separate criteria for case-control and cohort studies to assess participant selection, comparability groups, and exposure and outcome definitions. Studies are awarded stars for meeting the criteria, and a higher number of stars indicates a higher quality study design, with a preference—evident by the potential of more stars—for cohort studies. Because our research aims do not favor a particular study design (i.e., our exposure of interest cannot be randomized or manipulated, cannot be caused by the outcome, and is unlikely to be subject to recall bias) or research question, we combined the study-specific scales and removed the criteria specific to each study design. This gives all study designs the potential to achieve the same number of stars and, thus, be considered high quality.
